# Cervicothoracic Multisegmental Transpinal Evoked Potentials in Humans

**DOI:** 10.1371/journal.pone.0076940

**Published:** 2013-10-07

**Authors:** Jonathan Einhorn, Alan Li, Royi Hazan, Maria Knikou

**Affiliations:** 1 The Graduate Center, City University of New York, New York, New York, United States of America; 2 Sensory Motor Performance Program, Rehabilitation Institute of Chicago, Chicago, Illinois, United States of America; 3 Department of Physical Medicine and Rehabilitation, Northwestern University Feinberg School of Medicine, Chicago, Illinois, United States of America; University of Sydney, Australia

## Abstract

The objectives of this study were to establish the neurophysiological properties of the transpinal evoked potentials (TEPs) following transcutaneous electric stimulation of the spine (tsESS) over the cervicothoracic region, changes in the amplitude of the TEPs preceded by median nerve stimulation at group I threshold, and the effects of tsESS on the flexor carpi radialis (FCR) H-reflex in thirteen healthy human subjects while seated. Two re-usable self-adhering electrodes, connected to function as one electrode (cathode), were placed bilaterally on the clavicles. A re-usable electrode (anode) was placed on the cervicothoracic region covering from Cervical 4 – Thoracic 2 and held under constant pressure throughout the experiment. TEPs were recorded bilaterally from major arm muscles with subjects seated at stimulation frequencies of 1.0, 0.5, 0.33, 0.2, 0.125, and 0.1 Hz, and upon double tsESS pulses delivered at an inter-stimulus interval of 40 ms. TEPs from the arm muscles were also recorded following median nerve stimulation at the conditioning-test (C-T) intervals of 2, 3, 5, 8, and 10 ms. The FCR H-reflex was evoked and recorded according to conventional methods following double median nerve pulses at 40 ms, and was also conditioned by tsESS at C-T intervals that ranged from −10 to +50 ms. The arm TEPs amplitude was not decreased at low-stimulation frequencies and upon double tsESS pulses in all but one subject. Ipsilateral and contralateral arm TEPs were facilitated following ipsilateral median nerve stimulation, while the FCR H-reflex was depressed by double pulses and following tsESS at short and long C-T intervals. Non-invasive transpinal stimulation can be used as a therapeutic modality to decrease spinal reflex hyper-excitability in neurological disorders and when combined with peripheral nerve stimulation to potentiate spinal output.

## Introduction

Epidermal or implanted electro-magnetic stimulation of the spine produces multisegmental compound muscle action potentials, termed here transpinal evoked potentials (TEPs), in distal and proximal muscles of upper and lower limbs in animals and humans [1-13]. TEPs, recorded from the soleus muscle, are facilitated following voluntary contraction of the homonymous muscle and depressed upon Achilles tendon vibration [2], suggesting that TEPs are modulated by spinal interneuronal circuits that mediate presynaptic inhibition. Further, the TEPs of the leg muscles are not susceptible to homosynaptic (or post-activation) depression [12,13], a spinal inhibitory mechanism documented for the H-reflex in both animals and humans and ascribed to presynaptic inhibition related to the amount of released neurotransmitters by the previously activated Ia afferents [14-19]. The time course of the homosynaptic depression of the soleus H-reflex is similar to that observed in the flexor carpi radialis (FCR) H-reflex [20], suggesting absent homosynaptic depression asymmetry in human upper and lower limbs.

TEPs recorded from leg muscles are not susceptible to homosynaptic depression because they are largely due to antidromic activation of afferents and orthodromic activation of motor axons [12,13], in opposition to the H-reflexes, which reflect the efficacy of monosynaptic activation of alpha motoneurons by primary spindle Ia afferents [21]. This neurophysiological property makes transcutaneous electric stimulation of the spine (tsESS) and associated TEPs suitable for diagnostic and/or therapeutic purposes in central nervous system neurological disorders, since TEPs bypass the pathological excitability state of alpha motoneurons. However, for this to be possible, a better understanding of the neurophysiological properties of TEPs as well as the neuronal pathways that tsESS is channeled in the human spinal cord is needed.

We have recently shown that transcutaneous magnetic or electric stimulation of the thoracolumbar spine attenuates significantly the soleus H-reflex, while the TEPs recorded from ankle and thigh muscles are not susceptible to homosynaptic depression in seated healthy subjects [12,13]. In this study, we examined whether similar effects are produced following transcutaneous electric stimulation of the cervicothoracic region. Accordingly, we established the susceptibility of TEPs recorded simultaneously from arm muscles to homosynaptic depression, the relationship of their latency and shape to FCR H-reflexes, the interaction of TEPs with peripheral (median) nerve stimulation, and the effects of tsESS on the FCR H-reflex in healthy people while seated.

## Materials and Methods

### Subjects

The experimental protocol was approved by the City University of New York (NY, USA) Institutional Review Board (IRB) committee (IRB No. 295205-4) and was conducted in compliance with the Declaration of Helsinki. Each subject signed an informed consent form before participating to the study. Thirteen adults (8 male, 5 female) free of any neuromuscular or orthopaedic disorders and between the ages of 23-42 years (26.54 ± 4.94; mean ± SD) participated in the study. In each subject, the blood pressure was monitored periodically during testing. No changes in blood pressure were observed during and after the experiment and no adverse effects were reported by the subjects.

### Electromyographic (EMG) recordings

Following standard skin preparation, single differential bipolar surface EMG electrodes (Bagnoli 8 system, Delsys, Boston, MA, USA) were placed bilaterally on the FCR, extensor carpi radials (ECR), biceps brachii (BIC), and triceps brachii (TRIC) muscles, and were secured with 3M Tegaderm transparent film (3M, St. Paul, MN, USA). All EMG signals were filtered with a cut-off frequency of 20 - 1000 Hz (1401 plus running Spike 2; Cambridge Electronic Design, Cambridge, UK) and were sampled at 2000 Hz.

### Transcutaneous electric stimulation of the cervicothoracic spine

Two re-usable self-adhering electrodes of 10.16 × 5.08 cm (cathode; model EP84169, Uni-Patch, MN, USA), connected to function as one electrode, were placed on the left and right clavicles. The Cervical 4 vertebra was identified through palpation, and a monopolar stainless-steel circular handheld electrode (anode) was used to establish the most optimal stimulation site. This site corresponded to the one that TEPs in most arm muscles could be evoked at low stimulation intensities. The monopolar hand-held stainless steel electrode was then replaced by a self-adhering electrode of 10.16 × 5.08 cm (model EP84169, Uni-Patch, MN, USA), and held under constant pressure throughout the experiment via an athletic wrap. The anode electrode was placed on top of the spine being equally on the left and right sides. Because of its size, the stimulating electrode covered from Cervical 4 to Thoracic 2 vertebrae levels. The anode and cathode electrodes were connected to a stimulator (DS7A, Digitimer, Welwyn Garden City, Hertfordshire, UK), that was triggered by an analog-to-digital acquisition system with customized scripts written in Spike 2 with single pulses of 1-ms duration. The stimulation intensity during which TEPs in the arm muscles were first noted on the oscilloscope (TDS 2014, Tektronix, Beaverton, OR, USA) was termed as TEP threshold. Stimulation intensities at TEP threshold ranged from 77 to 228 (159.15 ± 54.69; mean ± SD) mA across subjects, while stimulation during testing was delivered at 1.2 (± 0.58) times the TEP threshold across subjects. At these stimulation intensities, subjects reported no pain or discomfort, while even at higher stimulation intensities no pain or discomfort was reported while a synchronous contraction of paraspinal and diaphragm muscles was evident.

### Median nerve stimulation (FCR H-reflex)

The FCR H-reflex was evoked by percutaneous stimulation of the right median nerve with rectangular shocks of 1-ms duration at 0.2 Hz delivered by a constant current stimulator (DS7A, Digitimer, Hertfordshire, UK). A hand-held bipolar stainless steel electrode was placed medial to the brachial artery on the cubital fossa, and used as a probe to determine the most optimal stimulation site [21]. This site corresponded to the one during which at low stimulation intensities Ia afferents could selectively be excited with absent activation of motor axons (M-wave), and the shape of the M-wave was similar to that of the H-reflex at both low and high stimulation intensities. When the optimal stimulation site was identified, the bipolar electrode was maintained in place via an athletic wrap. The median nerve was stimulated at intensities that ranged from 28 to 331 (122.62 ± 74.49) mA across subjects.

### Experimental protocol

With the subjects seated and having established the most optimal stimulation sites, TEPs in the arm muscles following tsESS were recorded randomly at 1.0, 0.5, 0.33, 0.2, 0.125, and 0.1 Hz in order to establish their susceptibility to homosynaptic depression. Then, a double tsESS pulse at an inter-stimulus interval of 40 ms, repeated once every 5 s, was delivered in order to establish whether the TEPs evoked by the second tsESS pulse are depressed compared to those evoked by the first tsESS pulse. At each stimulation frequency (from 1.0 to 0.1 Hz) and in the double tsESS pulses paradigm, 10 TEPs were recorded.

Then, the maximal M-wave following median nerve stimulation was evoked and measured as peak-to-peak amplitude on the digital oscilloscope and saved for offline analysis. The stimulation intensity was adjusted to evoke FCR H-reflexes on the ascending limb of the recruitment curve that ranged from 15 to 25% of the maximal M-wave or equivalent to 50% of the maximal FCR H-reflex. A double pulse at an inter-stimulus interval of 40 ms, repeated once every 5 s, was delivered to the median nerve in order to establish whether the FCR H-reflexes evoked by the second pulse are depressed compared to those evoked by the first pulse. In order to establish the effects of peripheral nerve stimulation on the TEPs amplitude, tsESS was preceded by median nerve stimulation at the conditioning-test (C-T) intervals of 0, 2, 3, 5, 8, and 10 ms. Further, in order to establish the effects of tsESS on spinal reflex excitability, the FCR H-reflex was conditioned by tsESS at C-T intervals that ranged from -10 (-10, -8, -3, and -2) to 50 ms (0, 2, 3, 5, 8, 10, 20 and 50 ms) with the subjects seated. Negative C-T intervals denote that tsESS was delivered after median nerve stimulation. Conditioned and control H-reflexes were randomly recorded, while for each set 20 H-reflexes at 0.2 Hz were recorded in all subjects.

### Data analysis

**Figure 1:**
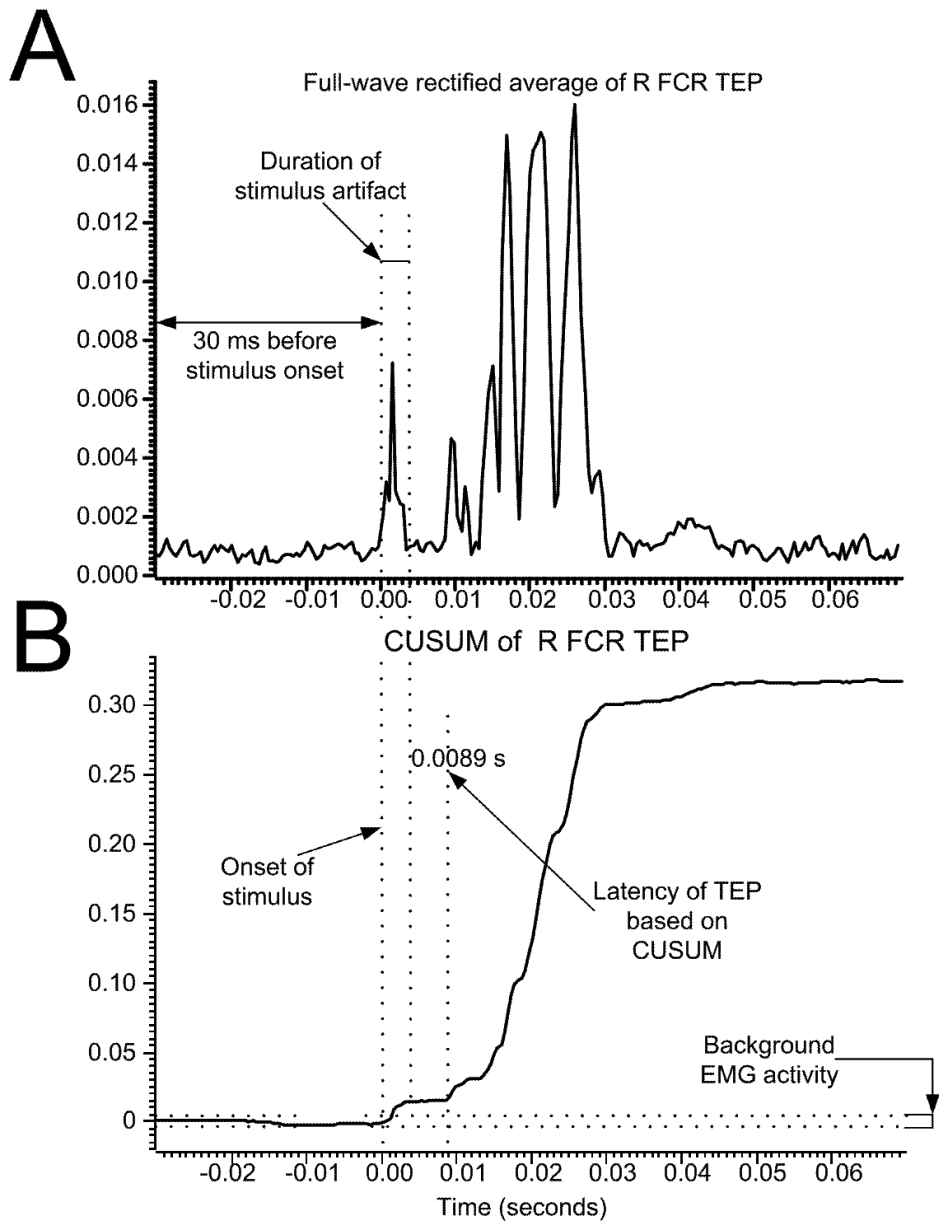
Latency calculation technique. Full-wave rectified waveform average (**A**) and associated cumulative sum (CUSUM) (**B**) characteristics of the TEPs (*n* = 10) elicited following non-invasive cervicothoracic transpinal stimulation and recorded from the right FCR muscle using surface EMG electrodes from one subject while at rest. The CUSUM latency, which represents the TEP latency, was determined based on the turning point of the CUSUM after the stimulus artifact.

Offline data analysis started with identification of the recorded muscle action potentials with a customized Spike 2 script. The latency of TEPs and FCR H-reflexes was estimated based on the cumulative sum (CUSUM) calculations by defining the precise turning point post-stimulus while taken into consideration the pre-stimulus EMG [22] (Figure 1). The CUSUM calculations were applied to the full-wave rectified waveform average. Then, the onset latency was grouped separately across subjects based on the muscle, and a Kruskal-Wallis one-way analysis of variance (ANOVA) on ranks was conducted to establish statistically significant differences of TEPs latency across muscles. A t-test was also used to establish statistically significant differences between the latencies of the left and right TEPs. The size of all recorded muscle action potentials was defined as the area under the full-wave rectified waveform from the onset latency until the action potentials returned to baseline EMG values.

For each subject, the TEPs recorded at 1.0, 0.5, 0.33, 0.2, and 0.125 Hz were expressed as a percentage of the mean amplitude of the associated TEPs recorded at 0.1 Hz. TEPs evoked by tsESS were grouped across subjects based on frequency and muscle, and an ANOVA for repeated measures at 5 × 8 levels (5: frequency, 8: muscles) was conducted to establish statistically significant differences across stimulation frequencies and muscles.

The FCR H-reflex conditioned by tsESS was expressed as a percentage of the mean amplitude of the control FCR H-reflex. The mean amplitude of the conditioned FCR H-reflex from each subject was grouped based on the C-T interval and a one-way ANOVA was conducted to establish statistically significant differences. This analysis was conducted also for the M-waves which were expressed as a percentage of the maximal M-wave.

In the double stimuli paradigm, the right and left FCR and ECR TEPs evoked by the second tsESS pulse were expressed as a percentage of the associated TEP evoked by the first tsESS pulse. The same analysis was done for the FCR M-wave and FCR H-reflex upon double stimuli delivered to the median nerve. The mean amplitude of each response from each subject was grouped based on the type of the response and an ANOVA for repeated measures was conducted. All statistical tests were conducted at 95 % of confidence interval. Mean amplitudes are reported along with the standard error of means (SEM), unless otherwise stated.

## Results

**Figure 2:**
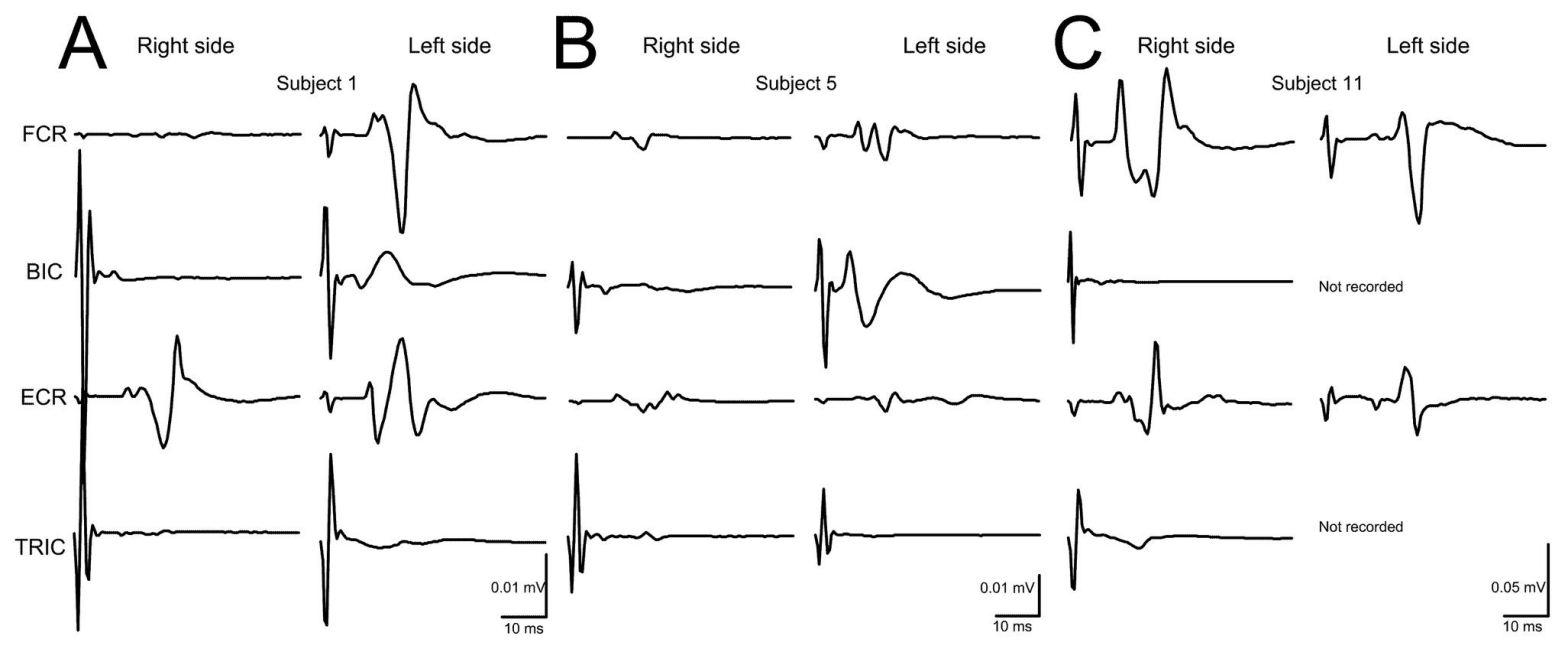
Waveforms of transpinal evoked potentials (TEPs). Representative examples of non-rectified waveform averages (*n* = 10, elicited at 0.2 Hz) of TEPs recorded following transcutaneous electric stimulation of the cervicothoracic region from the flexor carpi radialis (FCR), biceps brachii (BIC), extensor carpi radialis (ECR), and triceps brachii (TRIC) muscles in both arms.

In Figure 2, non-rectified waveform averages (*n* = 10 evoked at 0.2 Hz) of TEPs recorded from three subjects while seated following tsESS of the cervicothoracic region are indicated. Note that the shape of TEPs for the right and left muscles is different for each subject and across subjects, consistent to the shape of the TEPs we recently reported for the leg muscles [12,13]. TEPs of arm muscles were manifested mostly with a triphasic waveform, but a polyphasic waveform was also present (see left ECR in subject 1 and right ECR in subject 11 in Figure 2).

**Figure 3:**
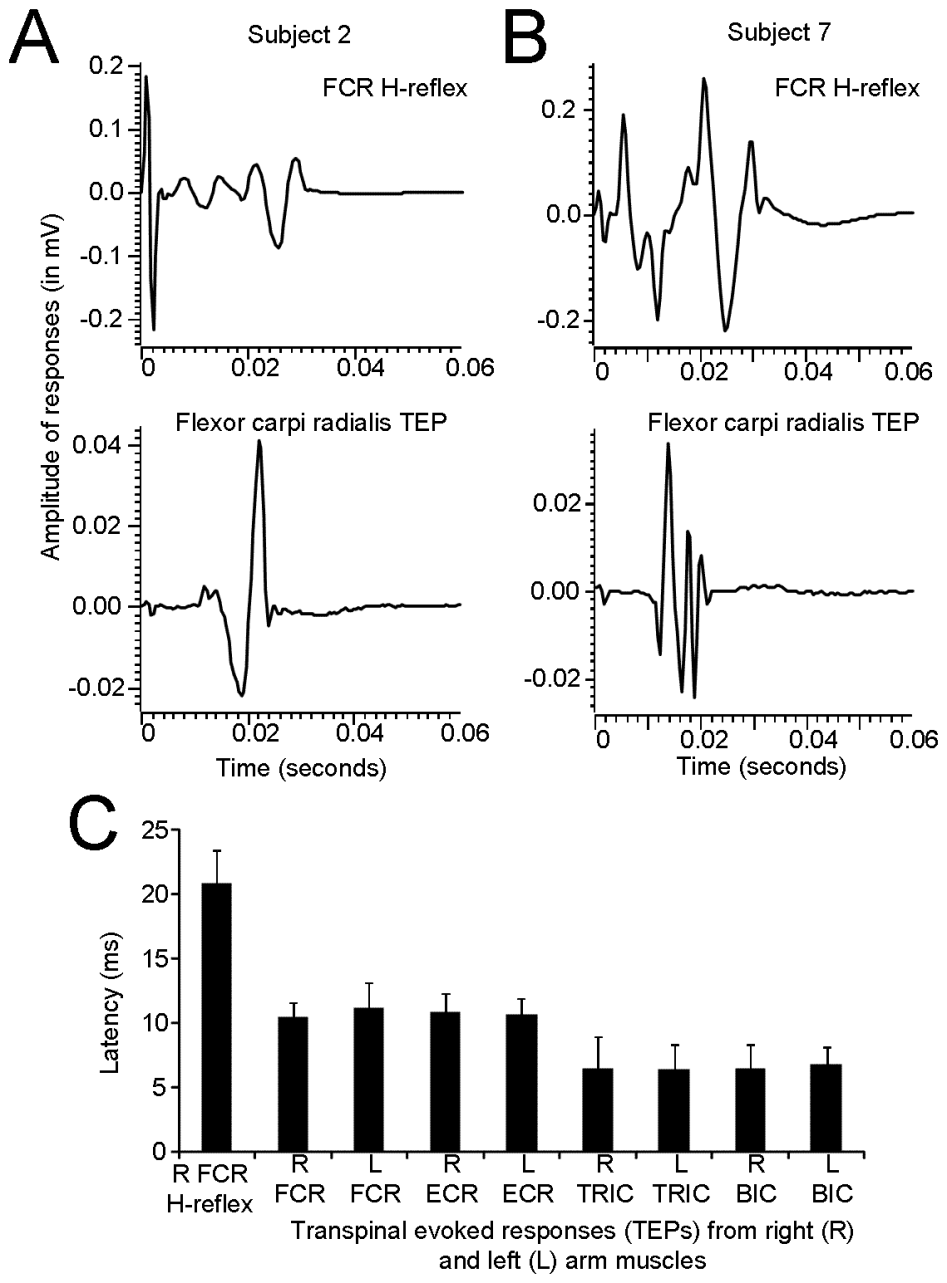
Latency of transpinal evoked potentials (TEPs). **A**, **B**: Non-rectified waveform averages (evoked at 0.2 Hz) of flexor carpi radialis (FCR) H-reflexes and FCR TEPs from two subjects. Note that in these subjects that the FCR TEP appeared nearly at a half latency compared to the FCR H-reflex latency. **C**: Overall mean latency of the FCR H-reflex and TEPs recorded from the left and right arm muscles following transcutaneous electric stimulation of the spine over the cervicothoracic region. Error bars represent the SD.

In Figure 3A and 3B, non-rectified waveform averages of the right FCR H-reflex and right FCR TEP from two subjects are illustrated. In subjects 2 and 7, the latency of the FCR H-reflex was 18.1 ms and 16.94 ms, respectively while the latency of the R FCR TEP was 11.4 ms and 10.2 ms, respectively. The mean latencies of the right FCR H-reflex and TEPs recorded from all muscles and subjects are indicated in Figure 3C. The FCR H-reflex latency (20.86 ± 2.5; mean ± SD) was 1.98 times the R FCR TEP latency (10.52 ± 1.06; mean ± SD), but the TEPs recorded from the ipsilateral antagonistic ECR and contralateral FCR and ECR muscles were prolonged by 0.43, 0.72, and 0.3 ms compared to the half latency of the R FCR H-reflex, respectively. A Kruskal-Wallis one-way ANOVA on ranks showed that the latency of the TEPs was statistically significant different from the FCR H-reflex latency (*F* (8)= 74.53, *P* < 0.001), while a *t*-test conducted separately for the pairs of the R and L FCR, ECR, TRIC, and BIC TEPs showed that the latencies were not statistically significant different (for all *P* > 0.05).

**Figure 4:**
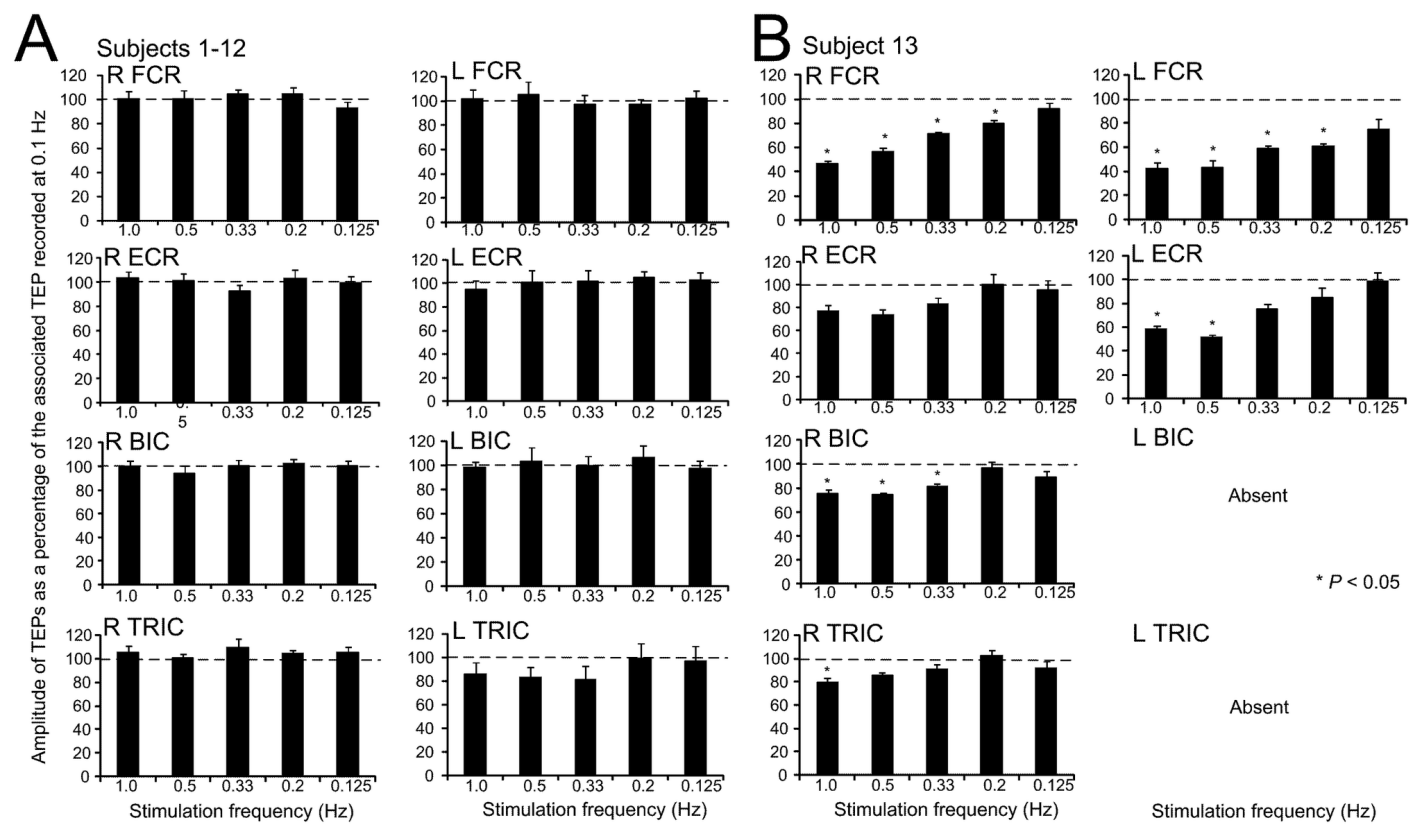
Susceptibility of transpinal evoked potentials (TEPs) to homosynaptic depression. The overall mean amplitude of the TEPs recorded bilaterally from the right (R) and left (L) flexor carpi radialis (FCR), extensor carpi radialis (ECR), biceps brachii (BIC), and triceps brachii (TRIC) muscles for subjects 1-12 (A) and for subject 13 (B). TEPs recorded at 1.0, 0.5, 0.33, 0.2, and 0.125 Hz are presented as a percentage of the mean amplitude of the associated TEPs recorded at 0.1 Hz. Asterisks indicate statistically significant differences from control TEPs values. Error bars represent the SEM.

The overall amplitude of the TEPs evoked by tsESS and recorded from the right and left FCR, ECR, BIC, and TRIC muscles at 1.0, 0.5, 0.33, 0.2, and 0.125 Hz is indicated in Figure 4. TEPs are presented as a percentage of the mean amplitude of the associated TEP recorded at 0.1 Hz. TEPs amplitude at different stimulation frequencies are grouped together for subjects 1-12 (Figure 4A), while data from subject 13 are shown separately (Figure 4B) because the effects observed in this subject were different from the other subjects and if grouped together these effects would have been masked. For data from subjects 1-12, the right FCR TEPs did not vary at different stimulation frequencies (*F* (5) = 2.94, *P* = 0.708; one-way ANOVA) (Figure 4A). The same result was also found for TEPs recorded from the other arm muscles. An ANOVA for repeated measures at 5 × 8 levels (5: frequencies, 8: muscles) showed that the amplitude of the normalized TEPs did not vary across muscles (*F* (7) = 1.74, *P* = 0.097) and across stimulation frequencies (*F* (4) = 0.59, *P* = 0.67), while a statistically significant interaction between muscles and stimulation frequencies was not found (*F* (28) = 0.38, *P* = 0.99). In contrast to these findings, in subject 13, the R FCR TEPs amplitude varied significantly across stimulation frequencies (*F* (5) = 44.97, *P* < 0.001; Kruskal-Wallis one-way ANOVA on ranks along with Dunn’s multiple comparisons), an effect that was observed in the L FCR, L ECR, R BIC, and R TRIC TEPs (Figure 4B).

**Figure 5:**
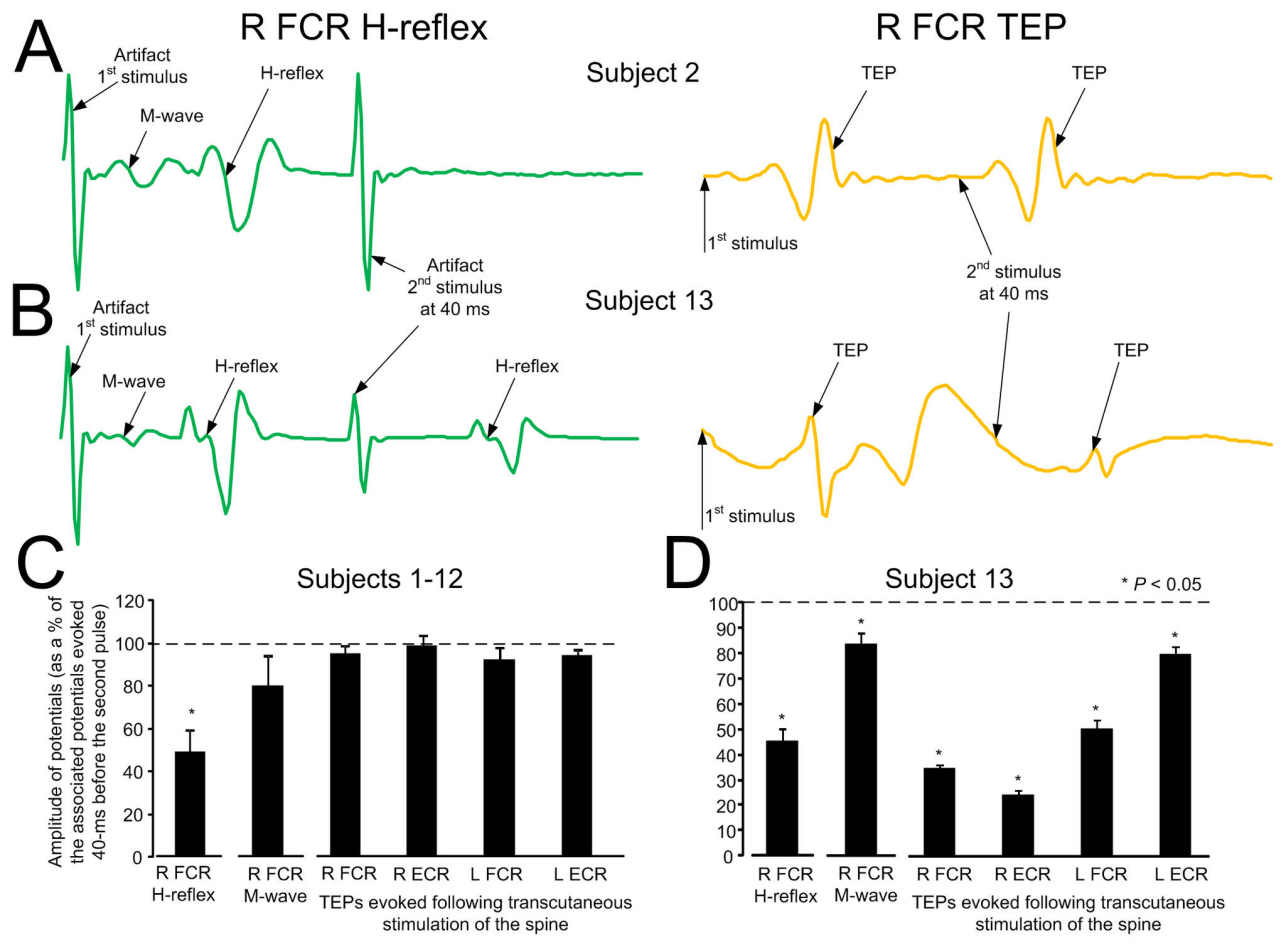
Transpinal evoked potentials (TEPs) and FCR H-reflexes upon double stimuli. **A**, **B**: Non-rectified waveform averages of the FCR H-reflex and the FCR TEPs when a double pulse at an inter-stimulus interval of 40 ms was delivered to the cervicothoracic region or to the median nerve, respectively for two subjects. **C**: Overall average amplitude of the FCR H-reflex, FCR M-wave, and TEPs for subjects 1-12 evoked by a second pulse delivered to the median nerve or to the cervicothoracic region. **D**: Overall average amplitude of the FCR H-reflex, FCR M-wave, and TEPs evoked by a second pulse delivered to the median nerve or to the cervicothoracic region for subject 13. In both C and D graphs, the FCR H-reflex, FCR M-wave and TEPs were normalized to the associated potentials evoked by the first pulse. Asterisks indicate statistically significant differences from control values. Error bars indicate the SEM.

In order to further delineate the susceptibility of TEPs to homosynaptic depression, TEPs and FCR H-reflexes were recorded following a double tsESS and/or median nerve stimulation pulse at an inter-stimulus interval of 40 ms. In Figure 5A and 5B, the effects of the double pulse on the right FCR H-reflex and FCR TEP are indicated. In subject 2, the FCR H-reflex was completely abolished following a second stimulus to the median nerve (Figure 5A, left graph), while the FCR TEP was not affected following a second tsESS stimulus (Figure 5A, right graph). In contrast, in subject 13, the FCR TEP was depressed following a tsESS pulse at 40 ms inter-stimulus interval (Figure 5B, right graph) similar to that observed for the FCR H-reflex (Figure 5B, left graph). The overall amplitude of the right FCR H-reflex, right FCR M-wave, and TEPs recorded from the right and left FCR and ECR muscles for subjects 1-12, elicited by a second stimulus and normalized to the mean amplitude of the associated action potential evoked by the first stimulus is indicated in Figure 5C. The FCR H-reflex amplitude was statistically significant different compared to the TEPs amplitudes (*F* (5) = 14.07, *P* = 0.015; one-way ANOVA) (Figure 5C). The mean amplitude of the right FCR H-reflex, right FCR M-wave, and TEPs recorded from the right and left FCR and ECR muscles in subject 13 are indicated in Figure 5D, which were all statistically significant different from the associated control values (*P* < 0.05). A one-way ANOVA showed that there was a statistically significant difference between the different types of responses (*F* (5) = 46.48, *P* < 0.001).

**Figure 6:**
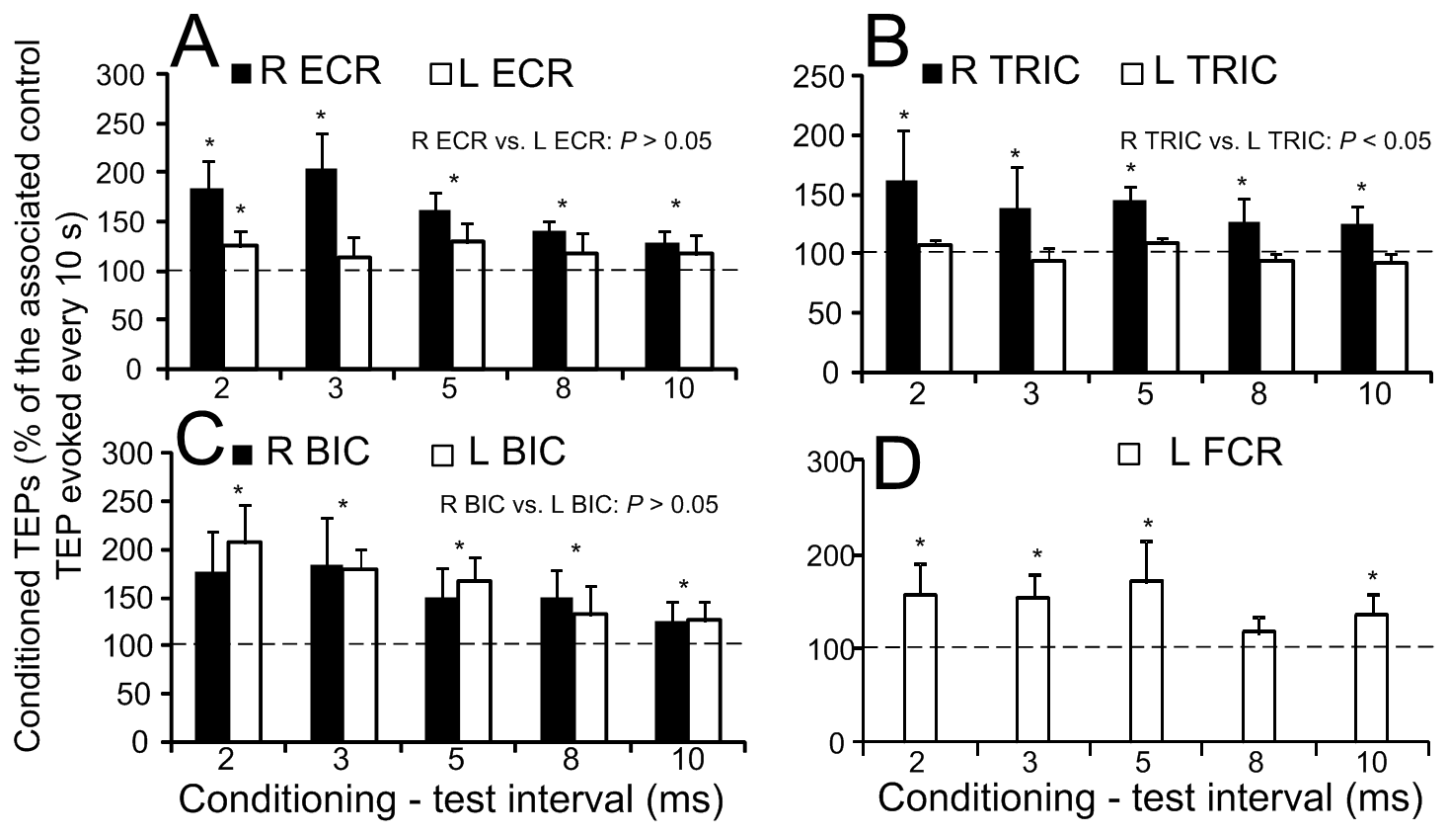
Effects of median nerve stimulation on the amplitude of transpinal evoked potentials (TEPs). Overall mean amplitude of the TEPs recorded bilaterally from the right (R) and left (L) extensor carpi radialis (ECR), triceps brachii (TRIC), biceps brachii (BIC), and left flexor carpi radialis (FCR) muscles following median nerve stimulation at low intensities (Ia afferent). On the abscissa the conditioning-test intervals (ms) tested are indicated. Asterisks indicate statistically significant differences of conditioned TEPs from control values. Error bars indicate the SEM.

The overall effects of median nerve stimulation on the TEPs amplitude recorded from the right and left ECR, BIC, TRIC muscles and from the left FCR are indicated in Figure 6. Note that TEPs for the R FCR muscle are not indicated because TEPs were contaminated by the stimulus artifact following median nerve stimulation and could not be measured accurately. Low-threshold median nerve stimulation increased significantly the TEPs amplitude recorded from the right ECR muscle when compared to control values at C-T intervals of 2, 3, 5, 8, and 10 ms (*F* (5) = 17.51, *P* = 0.004; Kruskal-Wallis one-way ANOVA on ranks along with Tukey’s test multiple comparisons) (Figure 6A). The same result was also found for TEPs recorded from the right TRIC (Figure 6B), right and left BIC (Figure 6C), and left FCR muscles (Figure 6D).

**Figure 7:**
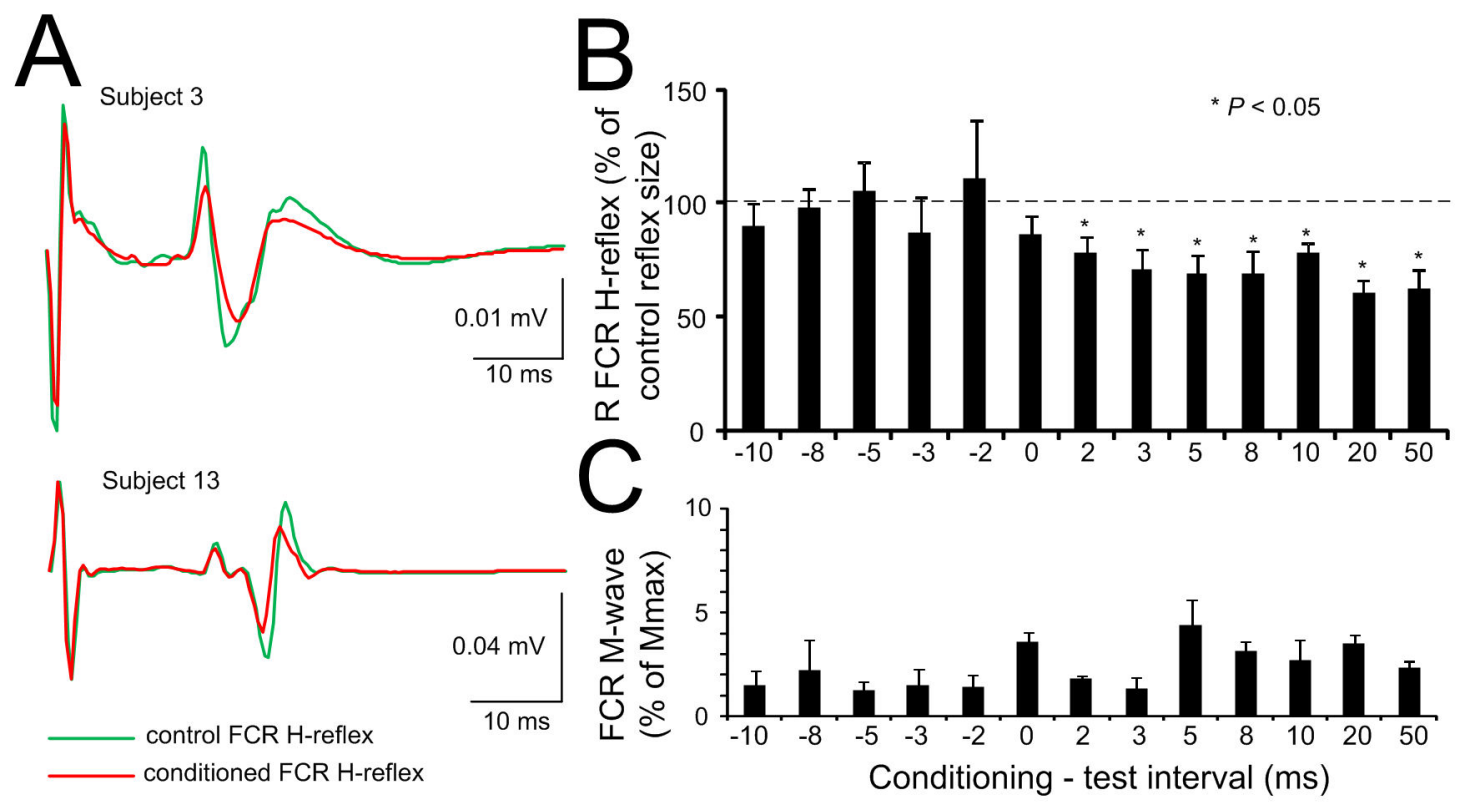
Effects of non-invasive cervicothoracic transpinal on FCR H-reflexes. **A**: Non-rectified waveform averages of the FCR H-reflex in two subjects under control conditions and following **transcutaneous electric stimulation of the spine (tsESS)** at the conditioning-test interval of 2 ms. Note that the FCR H-reflex depression occurs with stable M-waves. **B**: Overall average amplitude of the FCR H-reflex conditioned by tsESS as a percentage of the control H-reflex. Asterisks indicate statistically significant differences between the conditioned and the control FCR H-reflex. **C**: Overall average amplitude of the FCR M-wave. Error bars indicate the SEM.

While median nerve stimulation facilitated the ipsilateral and contralateral TEPs, tsESS of the cervicothoracic spine depressed the FCR H-reflex. The effects of tsESS at the C-T interval of 2 ms on the average soleus H-reflex recorded from two subjects while seated is shown in Figure 7A. In both examples, the magnitude of the conditioned FCR H-reflex was significantly reduced when compared to control reflex values, and the FCR H-reflex depression occurred with stable FCR M-waves under control conditions and during tsESS. The amplitude of the conditioned FCR H-reflex from all subjects and C-T intervals tested is shown in Figure 7B. The FCR H-reflex varied significantly across the C-T intervals tested (*F* (12, 65) = 2.09, *P* = 0.03), while the FCR H-reflexes at the C-T intervals ranging from 2 to +50 ms were statistically significant different from the other C-T intervals and from control reflex values (*P* < 0.05). The FCR H-reflex depression coincided with stable M-waves (*F* (12) = 2.4, *P* = 0.992; one-way ANOVA) (Figure 7C).

## Discussion

Transcutaneous electric stimulation of the spine over the cervicothoracic region attenuated significantly FCR H-reflex excitability in seated healthy human subjects, while TEPs recorded from arm muscles were not susceptible to homosynaptic depression in all but one subject, and were facilitated following peripheral nerve stimulation. These findings are consistent to those we have recently reported for TEPs recorded from the leg muscles following electric or magnetic stimulation of the thoracolumbar region [12,13], supporting that neuronal pathways associated with TEPs and tsESS-mediated reflex effects in human upper and lower limbs are similar. The neurophysiological characteristics of the TEPs and the use of tsESS as a therapeutic modality are discussed in the following sections.

TEPs were present at similar latencies in left and right arm muscles with shorter latencies observed for the biceps/triceps muscles (L BIC: 6.8 ± 1.29 ms) compared to the more distal wrist flexors/extensors (R FCR: 10.5 ± 1.06 ms) (Figure 3B), consistent to that reported elsewhere [11,23]. It should be noted that the R ECR, L FCR, and L ECR TEPs latency was prolonged by ^~^0.5 ms when compared to the half latency of the R FCR H-reflex, supporting for synaptic events between antagonistic muscles and interlimb neuronal circuits upon manifestation of TEPs. Across the studies reported in the literature, the latency of the FCR TEPs ranges from 7.17 to 8.46 ms [11,23], while TEPs recorded from thenar muscles following transcutaneous magnetic stimulation over the cervicothoracic spine had an average latency of ^~^13.5 ms [24]. Differences of latencies reported in the literature may be related to the placement and size of the stimulating electrode, anthropometric characteristics, as well as to the thickness of the underlying tissue and the methodology utilized to evoke and measure the latency of these potentials [2,25,26].

The shape of the TEPs following tsESS of the cervicothoracic region was largely triphasic in both wrist and elbow flexor/extensor muscles (Figure 2), a shape apparent for the FCR H-reflex (Figure 2, Figure 3A, Figure 5A, B). Further, the shape of the TEPs was asymmetrical across muscles for the same subject throughout the whole duration of an experiment (Figure 2). The different shape of TEPs across muscles within a single subject suggests that TEPs recorded from different muscles might be due to different synaptic events and thus have different origin, in a similar manner to the spinal segmental potentials recorded following stimulation of the posterior tibial nerve [27]. TEPs may be due to concomitant excitation of different types of afferents and motor axons, and thus their waveform may contain a direct motor response (i.e., M-wave) as well as reflex-mediated action potentials. If TEPs encompass an M-wave, then they should not be influenced by repetitive discharges, while their amplitude should decrease when reflex-mediated synaptic events are present upon their manifestation. This constitutes a clear distinction between a motor response and a reflexly-afferent mediated response, since the FCR H-reflexes are depressed after a preceding homonymous reflex discharge occurring at 1-2 s [20]. In this study, TEPs recorded from arm flexor/extensor muscles were not affected when stimulation was delivered at low-frequencies in all but one subject (Figure 4), supporting that they are not susceptible to homosynaptic depression and likely do not represent reflexly-mediated action potentials. This is consistent to our recent findings reported for TEPs of the leg muscles in healthy subjects [12,13]. In order to elucidate further this neurophysiological property, we recorded FCR H-reflexes and TEPs upon double stimuli delivered at an inter-stimulus interval of 40 ms. The FCR H-reflexes were clearly depressed upon double stimuli reaching an amplitude half of the responses obtained with the first stimuli (Figure 5C, D), while the amplitude of the TEPs was not significantly affected (Figure 5C). However, in subject 13, the right and left FCR and ECR TEPs were depressed upon double stimuli, consistent to the depression of TEPs observed in this subject following tsESS at low frequencies (Figures 4B, 5D). The depression of the TEPs across muscles upon double stimuli and at low stimulation frequencies in this subject suggests that these potentials were affected by homosynaptic depression and thus we can propose that in this subject TEPs were partly reflexly-mediated evoked potentials involving activation of group I afferents. While we cannot know why TEPs in this subject were channeled into the spinal cord through reflex pathways, it is possible that this effect may be related to a different orientation of the stimulating electrode relative to the cervical roots and trunks of the brachial plexus compared to the other subjects, because the paraspinal muscles of this subject were not hypertrophic as his life style was sedentary. Because F-waves are depressed upon double stimuli due to refractoriness of motor axons by the preceded afferent volley [28], one may consider that TEPs in subject 13 are F-waves, which are produced at high stimulation intensities of a mixed peripheral nerve and are recurrent discharges of antidromically activated motoneurons [29]. However, TEPs cannot be considered to reflect F-waves because the TEPs latency of thenar muscles is shorter by 1.4 ms when compared to F+M-1/2 latency [30], and the latency of TEPs in this subject was similar to that observed in the other subjects. In conclusion, TEPs of arm muscles following stimulation of the spine at the cervicothoracic region represent composite excitatory potentials of motor nerve fibers excited orthodromically and different types of afferents excited antidromically in the majority of subjects, while in some subjects TEPs may represent excitation of spinal reflex pathways similar to that known for the H-reflexes in humans.

Most notable findings of this study are the FCR H-reflex depression following tsESS (Figure 7B) and the facilitation of TEPs recorded from left and right arm muscles following percutaneous stimulation of the right median nerve (Figure 6). These findings have never been documented in humans and have great clinical significance. The FCR H-reflex depression is in line with the soleus H-reflex depression we reported following transcutaneous electric or magnetic stimulation of the thoracolumbar spine [12,13]. FCR H-reflex depression might have been mediated at a presynaptic or postsynaptic level or simultaneously at both synaptic levels. Potentiation of Ia afferents hyperpolarization by antidromic excitation of group Ia afferents in the dorsal columns [5], repetitive volleys produced by activation of fast conducting afferents acting at a postsynaptic level [31], polysynaptic spinal reflex pathways affecting alpha motoneurons [23], and supraspinal activity modulated by the orthodromic transmission of impulses in dorsal column fibers following tsESS may all account for the FCR H-reflex depression at short and long C-T intervals. Because in anaesthetized rats, spinal direct current stimulation modulates in a polarity-specific manner the supraspinal activity of the somatosensory system [32], and spinal cord neurons excitability [33], both supraspinal and spinal neuronal circuits might have mediated the FCR H-reflex depression observed in this study.

While the FCR H-reflex was depressed following tsESS, TEPs recorded from the left and right arm muscles were facilitated at C-T intervals ranging from 2 to 10 ms following stimulation of the right median nerve (Figure 6). The aforementioned mechanisms for FCR H-reflex depression cannot account for the facilitation of TEPs. Stimulation of the median nerve produced action potentials that run dorsally and perpendicular to the surface of the cord representing activity of dorsal roots travelling rostrally and ventral roots travelling caudally [34]. These spinal potentials could have modulated activity of interneurons that are involved in interlimb coordination, supported by the fact that facilitation of TEPs was observed on both arms. However, changes in the excitability of the stimulated axons cannot be disregarded. Based on the current experimental protocol, the exact neuronal pathway cannot be determined, and it is apparent that further research outlining facilitatory and inhibitory synaptic events, including but not limited to motor unit recordings and peristimulus frequencygrams [35], associated with TEPs is needed.

### Clinical application of findings

Invasive dorsal column stimulation in two people with multiple sclerosis improved their motor, reflex, and bladder function [36], while continuous epidural stimulation enabled a person with motor complete paraplegia to achieve full-weight bearing and locomotor-like EMG activity [37], consistent to the modulation of spinal locomotor networks of adult spinal rats [38]. These results were obtained with stimulation administered invasively, limiting thus their possibility of application to a larger number of patients. This study showed for the first time that non-invasive transpinal stimulation of the cervicothoracic region depressed FCR H-reflex excitability, and when cervicothoracic transpinal stimulation was combined with peripheral nerve stimulation the spinal output - manifested as multisegmental TEPs - was facilitated. Based on our current and published findings [12,13], tsESS can be utilized in upper motor neuron lesions to normalize the reflex hyper-excitability of upper and lower limbs, and to potentiate spinal output.
